# Carotenoids: Potent to Prevent Diseases Review

**DOI:** 10.1007/s13659-020-00244-2

**Published:** 2020-05-13

**Authors:** Takshma Bhatt, Kirtan Patel

**Affiliations:** Department of Biotechnology, President Science College (Affiliated to Gujarat University), Ghatlodia, Ahmedabad, 380061 India

## Abstract

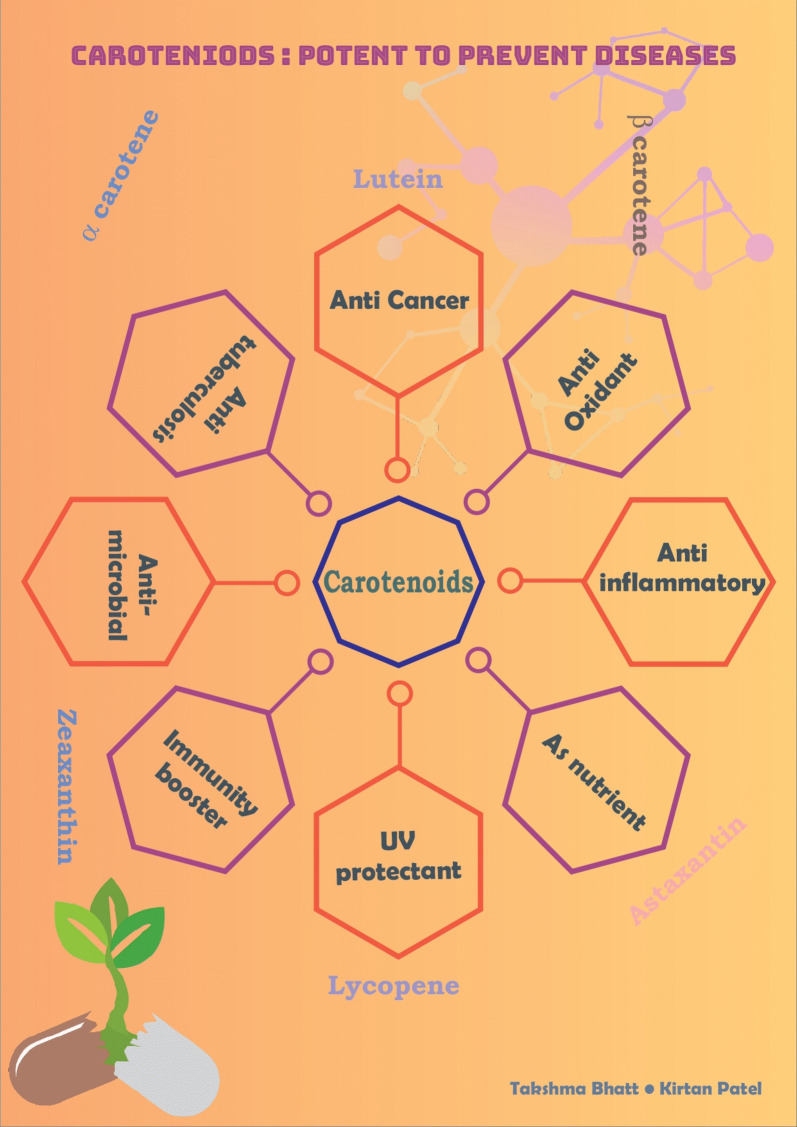

## Introduction

**Fig. 1:**
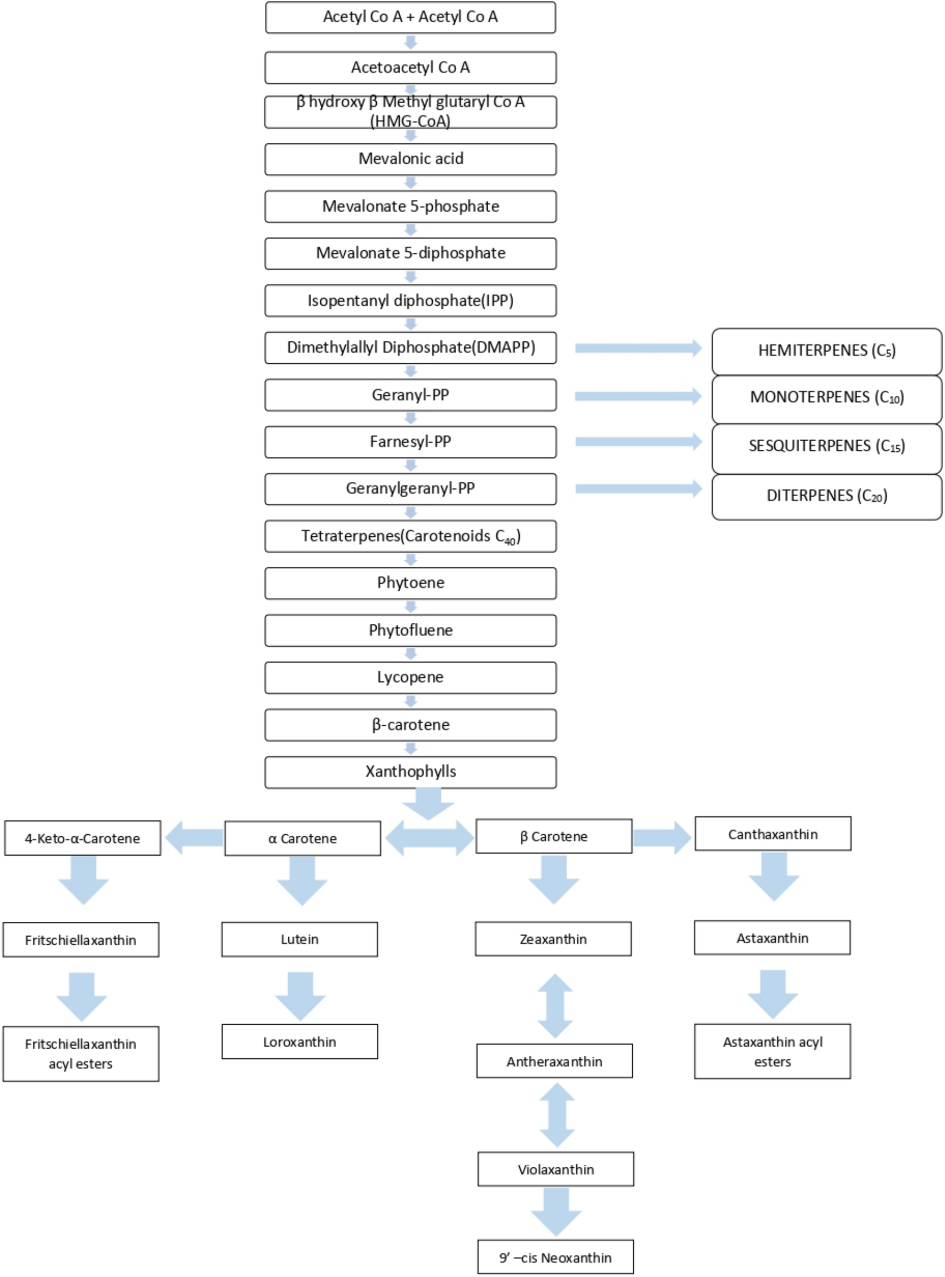
Biosynthesis pathway of carotenoids [1, 7]

Carotenoids are fat-soluble, highly unsaturated red, orange, or yellow pigments that are naturally present in plants, fungi, bacteria, and algae, where intensity of colour is generally related with the number of carotenoids. Carotenoids are naturally found in abundance in vegetables and fruits. Moreover, certain photosynthetic bacteria and algae are also the good source of these compounds [1]. The phytochemical carotenoids belong to the isoprenoids and their basic structure is made up of eight isoprene units, having C 40 backbone. Majorly, two types of carotenoids can be discerned: Carotenes-the pure hydrocarbons while xanthophylls are derivatives that contain one or more oxygen functions [2]. Carotenoids collaborate with other biomolecules such as proteins and lipids to enhance its activity as anti-oxidant [3]. A noticeable function of this phytochemicals in plants are to protect the cells from extra UV light mainly not useful for photosynthesis as it induces stress on the plant cells hence, here they show their antioxidant property.Moreover, these carotenoids gets cleave into apocarotenoids which is responsible for aroma, colour and phytohormone production. It also helps in producing signals among the plant cells [4] (Fig. 1).

## Classification and Biosynthesis

Carotenoids are classified into two groups: (i) the compound having single long carbon chain known as carotenes (ii) the compound having oxygen atom in its structure is known as xanthophylls. These carotenoids are further divided into the category of having provitamin A activity that is they are able to characterize further into vitamin A. Due to the lipophilic and hydrophobic nature they can be easily extracted from natural sources like green vegetables, flowers, fruits and from microorganisms. Methods used to extract carotenoids are: Supercritical fluid extraction, Solvent extraction and many other [5].

**Fig. 2:**
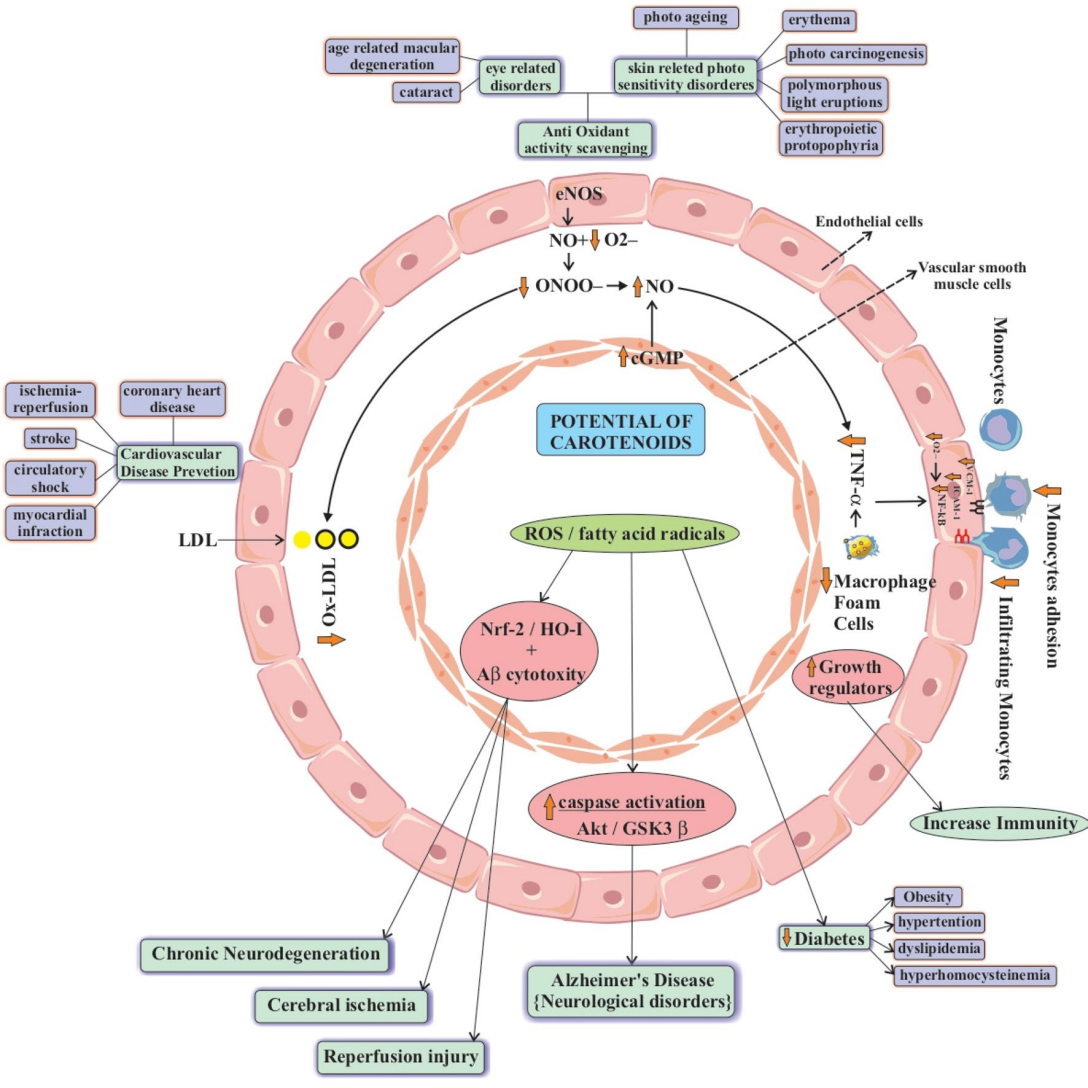
Potential of carotenoids to prevent diseases [18, 31]

Biosynthesis of carotenoids have been studied on various vascular plants and on microorganisms from which the pathways of its occurrence came to existence known as carotenogenesis.Carotenogenesis is of five stages (i) the active isoprene are formed through isoprenoids building blocks (ii) condensation of isoprene units resulting in forming phytoene (iii) formation of lycopene through extension by four desaturation steps and isomerization (iv) cyclization of lycopene ends to form carotenes (v) involvement of oxygen to form various xanthophylls [1, 2] (Fig. 2).

## Properties

### Anti-oxidation

Mainly diseases are caused due to free moving oxygen radicles in the body. Diseases like cancer, cardiovascular diseases, ophthalmic diseases, neurodegenerative diseases are caused due to free radicles [6]. Carotenoids are able to scavenge the free oxygen radicals from the body which helps in curing certain types of cancer and reduce the formation tumour in cancer patients. Moreover, certain carotenoids are found to activate the antioxidant gene expression through Nrf2 (Nuclear factor erythroid 2 related factor 2) transcriptional factor which helps in decreasing neurological disorder and diabetes [7, 8]. Another study has reported that carotenoids extracted from *M. roseus* and *M*. *luteus* have significant antioxidant property showing (IC50 3.5–4.5 mg/mL) through radical scavenging assay of DPPH [9]. This can be obtained from the sources like spinach, banana, cabbage, carrot etc.

### Anti-inflammatory

Carotenoids having oxygen in structure like fucoxanthin and astaxanthin has proved to suppress the expression of cytokines IL-6, TNF-α and IL-1β and act as pro and anti-inflammatory compounds [10]. This can be understood by the figure where it is seen that as carotenoids scavenges the oxygen radicle, it will not further able to interact with NF-κB which results into macrophage foam cells and decrease in TNF-α [18, 31].

### Antibacterial and Anti-viral

In all over the environment billions of bacteria are present and many of them can able to cause chronic diseases and various kind of infections. Moreover, the natural organic pigments of carotenoids can able to combat against those pathogenic bacteria. The hundreds of research experiments show that antibacterial property. According to that the turbidity observations of minimal bactericidal concentration assay shows the lowest concentration of bacterial colonies on nutrient media before that for comparison and cross checking, the Minimal inhibitory concentration assay carried out, consequently 95 percent similarity observed and that study carried out through halobacterial carotenoids against antibiotic resistant microbes which are *Staphylococcus aureus*, *Klebsiella pneumoniae, Streptococcus epidermis, Pseudomonas aeruginosa* and *Streptococcus pneumonia*. Results observed were terrifying as some species were not able to grow under the presence of carotenoids [11]. Carotenoids has given rise to a new research on its use as anti-viral agent. Carotenoids have shown that it helps to fight against viruses. In an experiment on Herpes Simplex Virus Type 1(HSV-1) it was found that *D. salina* extract and *H. pluvialis* extract can reduce its activity ranging from 50 to 85% [12].

### Anticancer

Several experiments show the potential of carotenoids as anti-cancer agents. It is seen in most of cases that carotenoids arrest the cell cycle which is associated with down regulation of cyclin D1, cyclin D2, CDK4 and CDK6 expression. Consequently, it also up regulates GADD45 α, which inhibits the entry of cell into S phase [13]. Moreover, compounds like crocin and crocetin extracted from saffron showed the anti-metastasis properties like anti-migration, antiinvasive and anti non adhesive effects in combination on 4T1 cell line in breast cancer [14]. Carotenoids like β-cryptoxanthin and lycopene are found suppressing the NF-κB signalling pathway which is effective against lung cancer and prostate cancer [15]. β-carotene has found to have anti angiogenic activity that is it helps to halt the process of developing new blood vessels which is often seen in cancerous tumours [16].

### Cardiovascular Diseases (CVD)

The various experiments which are occurring inside of body and at lab facility have appeared that carotenoids diminish inflammation and oxidative stress by promoting normal mechanism of cell. In addition to its Many scientific studies also show that by increasing carotenoid rich food helps in reducing CVD in patients [17, 18] Carotenoids works straightforwardly by evacuating superoxide anion (O^2−^), in receptive oxygen species (ROS) generation, also have appeared to re-establish nitric oxide (NO) endothelial bioavailability. Consequently, they might be viewed as a potential source of oxidant modulators of endothelial reaction to pro-oxidant/inflammatory stimuli [18]. Certain carotenoids like astaxanthin, lutein and β cryptoxanthin are found more involved in preventing cardiovascular disease by oxidizing LDL and decreasing HDL. This can be used in treating myocardial injury and many more [19]. To prove other sources than plants, a carotenoid rich fraction of *D. salina* was taken and tested. Which proved that carotenoids can attenuate the cardiac dysfunction in obese rats [20]. Hence, it can be used as food additives to reduce the obesity associated cardiac dysfunction. Moreover, several critical studies from human are disputed and challenging. According to it especially in vivo experimental procedures for cardiovascular protection are not well known more work to be done is left [18].

### Ophthalmic Infections

Vitamin A plays a vital role in human’s eye as it is component of rhodopsin which facilitates the efficient transfer of energy from photos of light to electrochemical signals. Deficiency of the vitamin causes night blindness; this can be prevented by up taking carotenoids in appropriate amount leading to good vision [21]. As mentioned earlier only few carotenoids about 10% can further categorized as pro vitamin A and later in vitamin A [17]. Lutein and zeaxanthin are oxygenated carotenoids present in the macular region of retina responsible for sharp and detailed vision which also serves as filters for blue light from screens and scavenges the free radicle oxygen from retina [6, 22]. Moreover, they can also help to prevent cataract in eyes and with ageing macular degeneration can also be prevented [21].

According to the investigated value of MIC and MBC of carotenoids from *Halomonas* sp. (HQ 438,316) against antibiotic resistant and ophthalmic bacterial pathogens, there are several bacteria found which causes eye infections and might be cured by carotenoids [11]. In addition to it *Staphylococcus aureus* can infect cornea (keratitis) or the inner chambers of the eye (endophthalmitis) [23], *Escherichia coli* in Conjunctivitis [24], and *Streptococcus pyogenes* and *Pseudomonas aeruginosa* in blepharitis [24].

### Neurodegenerative Diseases

In nervous system increase of oxidative stress results into several neurodegenerative diseases such as Alzheimer’s, Huntington’s, Parkinson’s and amyotrophic lateral sclerosis (ALS). Several diseases are due to Ca^2+^ inability to signal the molecules but carotenoids like astaxanthin, βcarotene and lycopene are involved in Ca^2+^ ion transportation in brain,with proper dietary of carotenoids malfunction due to improper signalling can be reduced [25]. The ability to cross the blood brain barrier, and cell mitochondrial membrane with stability along with antioxidant property carotenoid-astaxanthin can be able to reduce the risk of diseases related to the nervous system (neurodegenerative). In addition to its Astaxanthin can combat neurodegenerative diseases by different properties such as anti-apoptosis, reduction in cerebral infarction in brain tissue, lowers ischemia by induced apoptosis, reduction of glutamate release and reduce free radical damage [25, 26]. Also it has been seen that lycopene makes blood brain barrier permeable, and it reduces when certain diseases occur [7]. In Alzheimer’s disease, ROS enhances caspase activation along with AKt/GSK-3β signalling. Carotenoids helps to bring this signalling normal and decreases caspase activation. ROS decreasing Nrf2/HO-1 or HO-1 with Aβ cytotoxicity results in cerebral ischemia or reperfusion injury and chronic neurodegeneration [8].

### Anti-hyperglycaemia

As indicated by the statistical analysis and of assessment of European Prospective Investigation into Cancer and Nutrition-Netherlands, from human utilization of carotenoids in their regimen can decrease the risk of type 2 diabetes. Notwithstanding it after the conclusive change of incorporated a few parameters like age, sexual orientation, risk factors, and diet, expressed that for beta carotene Hazard proportion found as 0.78 though for alpha-carotene, 0.85. Additionally, that studies demonstrate that usage of carotenoids can diminish the risk of diabetes type 2 for sound women and men [27]. Main reason of hyperglycaemia is the lifestyle and food habits. Due to hypertension oxidative stress is induced which results in complexity with body by associating it with obesity, diabetes, dyslipidaemia and hyperhomocysteinemia. Here, fatty acid radicles and reactive oxygen species play vital role in increasing the GR, GPx and other hormones in body leading to diseases. Carotenoids by scavenging this fatty acid radicle and ROS brings regulatory signals back to normal and reduce (40 to 79%) diseases [28, 29].

### UV Radiation Protection

Not only limited to anti-oxidant and anti-inflammatory, UV light protection can be also determined through various studies which in the stated experiment of Efficacy of UV‑C protective activity of carotenoid pigments isolated from *M. roseus* and *M. luteus* on growth of S. faecalis proved that the 21 colonies out of 31 colonies has been shown stable and strong resistant against UV exposure with approximately 70 to 95 percentage of coefficient of variation at 120 min [9]. In various carotenoids Beta-carotene and canthaxanthin have explicit photo defensive properties. In an experiment it indicated that reactive oxygen species and other reactive free radicals helps alongside in oxygen quenching, which respond in blood and skin erythropoietic protoporphyria patients. Moreover, erythropoietic protoporphyria patients have shown decreased level of β carotene in their serum for which they need to take it as dietary supplement [23, 30, 31] Moreover, it is also notices that lycopene and beta carotene helps to reduce skin redness and damage under UV rays [6] which can be helpful as soothing agent under UV rays of sun.

### Proliferating Agent

Major role of carotenoids differentiated into pro vitamin A is in cell differentiation and tissue growth [21]. Immunomodulating function has been observed while experimenting on spleen cells showing increased response to mitogens, from this it may be derived that carotenoids enhance the activity of natural killer cells [32]. Also it helps to boost immune system by rising cell to cell communication by increasing the exchange of growth regulatory signals this leads to apoptosis in damaged cells [6].

### Anti-tuberculosis

Nowadays world is fighting against Multidrug resistant (MDR) bacteria i.e. *Mycobacterium tuberculosis.* With a series of experiments on biomass of *C. vulgaris*, this come up with the new therapeutic application of carotenoids as anti-Tuberculosis agent. 100% of inhibition was observed at 100 μg/mL by fatty acids-carotenoids complexes (sample 1 red oil) and 50 μg/mL of (sample 2 brown oil) [33]. Much work is still needed in this field as this disease is related to oxygen and carotenoids are known for its antioxidant properties.

### Regenerative Liver

Consumption of fruits and vegetables and several biological reactions states different valuable properties along with antioxidant in regeneration (cure) of liver. Carotenoids are utilised in liver and lipoprotein component within the purpose of secretion into blood circulatory system. The utilised pigments of carotenoids combat oxidation mechanism during high level of free radical species in liver organ also these features prevent the growth of liver dysfunctionality [34–36]. Moreover several studies stated that b-carotene, lycopene, lutein, and b-cryptoxanthin types of carotenoids have antioxidant effects against lipid peroxidation in rat liver [35, 36]. Carotenoids are found connected with adipose tissue and multiple serum in humans for different metabolism such as insulin sensitivity in liver and adipose tissue [37]. In Most of the cases liver damage happens from the high value of cholesterol. A marine Astaxanthin (Carotenoid) can able to function as protection for cells, fats and other membrane proteins towards oxidative damage. Moreover, the intestinal cells partially allow Astaxanthin enter into chylomicrons. The Astaxanthin dissolved in chylomicrons through lipid enzymes are secreted from lymph to liver. In chylomicrons ROS will be quickly evacuated from different tissues as well as from liver. In that way amongst all carotenoids, Astaxanthin is one that can have ability to fight and cure liver [26].

### As Nutrient

Intake of higher total carotenoids has shown the benefit of reduction in fracture incidence by increasing bone density and has provided positive effect of bone mineral status at all age group which resulted in reduction of under carboxylated osteocalcin, hence regular intake of carotenoid rich foods may help in decreasing the osteoporosis in patients [38]. Norbixin an apocarotenoids, has found to have antioxidant and food additive application also helps in repairing ROS dependent DNA damage. Lycopene is used as food colouring agent and flavour modifier found from the natural source tomato [16] used commercially in making sauce, ketchup and as intensifier in food, pharmaceutical and dye industries [5].

## Conclusion

From the above discussion it can be said that this mighty molecules of nature has immense power to defeat many diseases as it not only prevents illness but also helps to cure them in certain cases. This carotenoids can be microencapsulated and can be commercialized as an antioxidant product, nutraceutical and pharmaceutical [16]. As world has come across to this hidden truth its demand have increased due to its properties of healing tissues and as a food additives (natural colourant) with nutritional value [5]. But much less is still know yet a far journey of research has to be taken to know carotenoid’s potential and power.

## Abbreviations

CDK4 and CDK 6: Cyclin dependent kinase 4 and 6
ALS: Amyotrophic lateral sclerosis
CVD: Cardiovascular diseases
decreasing HDL: Decreases high density lipoprotein
*D. salina*: *Dunaliella salina*
GADD45 α: Growth arrest and DNA-damage-inducible, alpha
GR, GPx: Glutathione reductase and glutathione peroxidase
GSK-3β/AKt: Glycogen synthase kinase-3β
HO-1: Heme oxygenase 1
HSV-1: Herpes simplex virus type 1
*H. pluvialis*: *Haematococcus pluvialis*
IL-6: Interleukin 6
MBC: Minimum bactericidal concentration
MDR: Multi drug resistant
MIC: Minimum (or minimal) inhibitory concentration
*M. roseus*: *Micrococcus roseus*
*M. luteus*: *Micrococcus luteus*
NF-κB: Nuclear factor kappa-light-chain-enhancer of activated B cells
NO: Nitric oxide
Nrf2: Nuclear factor erythroid 2 related factor 2
oxidizing LDL: Oxidized low-density lipoprotein
ROS: Receptive oxygen species
TNF-α: Tumor necrosis factor alpha
UV light: Ultra violet light
